# A Likely Diagnosis of Familial Partial Androgen Insensitivity Syndrome in Three 46, XY Siblings With Ambiguous Genitalia: A Case Series

**DOI:** 10.1002/ccr3.73470

**Published:** 2026-09-10

**Authors:** Tayyeb Ali, Muhammad Hassaan Javaid, Sibgha Fawad Memon, Zauha Fawad Memon, Munazza Iqbal, Sadia Afridi, Abdul Rahim Soroush, Adnan Iqbal Khan, Naseer Ahmad, Nayab Ayub Afridi, Zuhaib Ali

**Affiliations:** ^1^ Gomal Medical College Dera Ismail Khan Pakistan; ^2^ Shifa College of Medicine Islamabad Pakistan; ^3^ Peoples University of Medical and Health Sciences for Women Nawabshah Pakistan; ^4^ Khyber Girls Medical College Peshawar Pakistan; ^5^ Balkh University Mazar‐i‐Sharif Afghanistan; ^6^ Saidu Medical College Swat Pakistan; ^7^ Hayatabad Medical Complex Peshawar Pakistan; ^8^ Liaquat University of Medical and Health Sciences Jamshoro Pakistan

**Keywords:** ambiguous genitalia: Androgen receptor dysfunction, disorder of sex development: 46, XY DSD, familial androgen insensitivity, partial androgen insensitivity syndrome

## Abstract

Familial partial androgen insensitivity syndrome is a rare cause of 46, XY disorder of sex development. It includes marked phenotypic variability, even among siblings. Persistent undervirilization despite normal androgen levels should prompt early multidisciplinary evaluation and counseling, especially where genetic testing is limited.

## 1 Introduction

Androgen insensitivity syndrome (AIS) is a rare X‐linked disorder of sex development (DSD), characterized by partial or complete insensitivity to androgen due to abnormal encoding of the androgen receptor (AR) gene in individuals with a 46, XY karyotype. AIS represents a spectrum of conditions that include under‐virilization of the external genital organs driven mainly by androgen dysfunction despite high serum androgen levels [1, 2, 3]. AIS is classified into three subtypes based on the severity of receptor dysfunction: complete AIS (CAIS), partial AIS (PAIS), and mild AIS (MAIS) [3].

Although mutations in the gene encoding AR are considered the molecular basis for PAIS, genetic verification may not always be possible due to limitations in available resources. In such situations, the diagnosis of PAIS is presumed based on several criteria, including clinical manifestations, hormonal analysis, imaging studies, karyotyping, ruling out other disorders, and family history. Thus, wherever it is mentioned in this paper, PAIS will be used as a presumptive diagnosis rather than a genetically confirmed condition.

PAIS demonstrates a spectrum of external genital phenotypes, ranging from female genitalia to ambiguous or partially under‐virilized male genitalia [1, 4]. The variable presentation of AIS is related to the AR gene, which is expressed on the X chromosome and encodes the nuclear receptor that brings about the effects of testosterone and dihydrotestosterone (DHT) on the target tissues essential for sexual differentiation at puberty [5]. Features like micropenis, bifid or underdeveloped scrotum, hypospadias, and variable cryptorchidism are some of the symptoms that reflect residual AR function [6, 7]. Ambiguous genitalia leads to early clinical suspicion and requires multidisciplinary evaluation, including endocrine, genetic, and radiological profiling to differentiate it from other DSDs like 5α‐reductase deficiency [4].

Phenotypic variation differs significantly in PAIS, even within family members having identical AR mutations. This highlights the crucial role of modifying factors and the absence of correlation across genotype and phenotype [8]. Occurrence of PAIS in siblings is rare and represents diagnostic and social challenges, which often require multidisciplinary assessment, including hormonal, molecular, and imaging studies [9]. Early identification, along with genetic counseling, plays a critical role in guiding future reproductive planning.

Here we report a case of three male siblings, aged 9, 3, and 7 years, having ambiguous genitalia and a 46, XY male karyotype.

## 2 Case Presentation

Three male siblings aged 9, 7, and 3 years were evaluated for longstanding ambiguous genitalia and undervirilization. All three children were assigned male at birth and had been raised as boys. Ambiguity of the external genitalia was recognized at birth and persisted throughout childhood. The familial occurrence of similar genital abnormalities prompted further evaluation for a possible inherited 46, XY DSD.

## 3 Methods

### 3.1 Differential Diagnosis

The possible diagnoses considered were 5α‐reductase deficiency, testosterone biosynthesis defects, mixed gonadal dysgenesis, and others related to 46, XY disorders of sex development. The combination of 46, XY karyotype, normal levels of testosterone and dihydrotestosterone, high levels of gonadotropins, incomplete virilization despite treatment with testosterone, cryptorchidism bilaterally, and familial inheritance suggested probable PAIS. However, AR gene mutation testing was necessary for a definite diagnosis but could not be done due to financial constraints and logistical limitations.

### 3.2 Investigations

Initial physical examination revealed a small phallus and poorly developed scrotal folds. The testes were not palpable in the scrotum in any of the three siblings. Subsequent ultrasonographic evaluation demonstrated bilateral undescended testes in the inguinal region or near the internal inguinal rings. The size of the testes was small for age. Testicular volume on the right side for Sibling 1 (9 years old) was 1.2 mL while that on the left side was 1.0 mL, both lying in the inguinal canal. Right and left testicular volumes in Sibling 2 (7 years old) were 1.0 mL and 0.8 mL, respectively, both being in the inguinal canal. In Sibling 3 (3 years old), both testicles had a volume of 0.5 mL each and situated near the internal inguinal ring. No Müllerian structures were identified on imaging. The patients have been evaluated various times throughout their lives for ambiguous genitalia and undervirilization, which have persisted into childhood, suggesting a familial pattern associated with DSD.

On the current evaluation, the following were noted: The external genitalia showed a phallus that was smaller than what is expected in the majority of males, and the musculature appeared underdeveloped for age and sex, as is typical in males. A chromosomal analysis of all the siblings showed a karyotype of 46, XY, which is indicative of XY DSD. Current endocrine studies showed very high levels of gonadotropin secretion, such as FSH and LH. There were very high levels of LH and FSH, and serum testosterone and dihydrotestosterone (DHT) concentrations were not reduced and were interpreted according to the laboratory reference intervals applicable to the patients' age and pubertal status. Because all three patients were prepubertal, interpretation of androgen concentrations was performed cautiously and in conjunction with the clinical phenotype, gonadotropin concentrations, and other available investigations. According to Table 1, despite testosterone and DHT concentrations that were not reduced, all three siblings demonstrated marked undervirilization of the external genitalia. This discordance between circulating androgen concentrations and the degree of genital virilization raised clinical suspicion for impaired androgen action, making PAIS the leading presumptive diagnosis.

**TABLE 1:** Clinical and laboratory characteristics of the three siblings.

Parameter	Reference range	Sibling 1 (9 years)	Sibling 2 (7 years)	Sibling 3 (3 years)
Karyotype	*—*	46, XY	46, XY	46, XY
Luteinizing hormone (LH), IU/L	Pediatric: < 0.3 IU/L; Adult male: 1.7–8.6 IU/L	2.8	0.65	0.50
Follicle‐stimulating hormone (FSH), IU/L	Pediatric: 0.3–3.5 IU/L; Adult male: 1.5–12.4 IU/L	6.7	7.0	3.0
Testosterone^a^	Prepubertal: 7–40 pg/dL; Adult male: 300–1000 ng/dL	301 ng/dL	105 ng/dL^b^	19 ng/dL^b^
Dihydrotestosterone (DHT), pg/dL	Pediatric: < 30 pg/dL; Adult male: 300–850 pg/dL	507	345	36.5
Pubic hair (Tanner stage)	*—*	Stage II	Stage II	Stage I
Gynecomastia	*—*	Absent	Absent	Absent
Right testicular volume (mL)	*—*	1.2	1.0	0.5
Left testicular volume (mL)	*—*	1.0	0.8	0.5
Testicular location	*—*	Bilateral inguinal canal	Bilateral inguinal canal	Near the internal inguinal ring (bilaterally)

^a^ Testosterone reference ranges differ by units as shown.

^b^ Values reported in ng/dL.

### 3.3 Treatment

During the early years of childhood, the patients started testosterone therapy to encourage growth of the penis, although the response was rather poor. Repair of hypospadias was considered, although delayed because of the small size of the penis. Bilateral orchiopexy of all brothers was finally completed. Despite all the above measures and the continuing use of androgens, the patients showed only partial virilization compared to the typical milestones in male development. Counseling was done in terms of gender identity, support, and outcome. All three siblings continue to identify as male.

## 4 Outcome and Follow‐Up

There had been minimal additional virilization noted during follow‐up observations of stable Tanner stages. There was success in orchiopexy and hypospadias repair in the oldest brother, and no complications were noted post‐op. There is persistent elevation of LH and FSH levels, as would be expected in a condition with partial androgen resistance. Testosterone and DHT levels are now within the therapeutic ranges. The three siblings remain under regular clinical follow‐up at approximately 3–6‐month intervals, although the duration of follow‐up differs among the patients. Follow‐up assessments include growth, pubertal development, genital phenotype, gonadal status, and hormonal parameters.

## 5 Discussion

PAIS is a rare form of 46, XY DSD, resulting from dysfunctional AR signaling despite high or normal serum androgen levels [10, 11]. Our case report describes an unusual familial occurrence of PAIS in three siblings, all raised as males, with ambiguous genitalia and under‐virilization with normal levels of testosterone and DHT [12]. It is rare for PAIS to appear in several siblings, which reflects its complex nature and the real‐world challenges families face in understanding and managing it.

Variations in the phenotypes seen in these three patients, that is, micropenis, underdeveloped scrotal folds, hypospadias, and cryptorchidism, are similar to those seen in other reported cases of PAIS [13]. Another notable feature is that the patients continued to have persistent symptoms despite being given testosterone therapy, which makes a diagnosis of androgen insensitivity rather than androgen deficiency. Despite testosterone and dihydrotestosterone levels remained unaffected, the three siblings presented with significant undervirilization. Such disparity between serum androgens levels and virilization is indicative of androgen deficiency and hence makes PAIS less likely. Nonetheless, the lack of genetic evaluation does not rule out other disorders associated with 46, XY DSD [14].

Previous research has demonstrated that patients with AIS often show variable responses to exogenous testosterone depending on AR activity, mutation types, and other environmental factors [15]. Due to certain limitations, genetic testing could not be performed; however, the clinical, biochemical, and karyotypic findings strongly support a clinical diagnosis of PAIS; however, without AR gene sequencing, the diagnosis remains presumptive. Alternative causes of 46, XY DSD were considered, although the combination of familial occurrence, clinical phenotype, hormonal profile, imaging findings, and karyotype made PAIS the most likely diagnosis. This also shows the challenges faced in resource‐limited areas, where financial, cultural, or religious constraints may hinder genetic evaluation for ethical reasons.

The presence of similar 46, XY DSD findings in three siblings suggests the likelihood of a familial disease inherited from the parents and is consistent with the mode of X‐linked inheritance in AR‐associated AIS. However, since no molecular genetics investigations were carried out, it is impossible to identify the exact genetic cause underlying inheritance within this family. At the same time, the different manifestations in three brothers highlight the variability in the clinical presentation of AR syndromes. Thus, the familial aspect of the case may be considered circumstantial evidence of X‐linked PAIS [4, 16, 17].

Management of PAIS requires a multidisciplinary approach, which incorporates endocrinology, genetics, and urological care [18]. In this case, the patient was started on testosterone therapy to promote penile growth, but no satisfactory results were obtained. Eventually, orchidopexy and hypospadias repair were performed to achieve favorable outcomes [12].

Navigating things like gender identity and psychological well‐being is a crucial part of caring for patients with DSDs [19]. In our case, adequate counseling was given to help families better understand the condition and social challenges.

The suboptimal response to testosterone treatment noted in all three siblings reflects the pathophysiological features of PAIS. Since the fundamental issue is a receptor defect rather the amount of androgens produced, the administration of testosterone will usually lead to only a slight enhancement of the process of virilization, especially when there are moderate to severe receptor defects. Therefore, testosterone administration is more of an adjuvant treatment than a definitive treatment, and surgery followed by multidisciplinary care becomes an essential part of the treatment process.

The main problem with this case series is that molecular genetic testing was not performed. Since the genetic testing for the AR gene mutation would have been the only way to establish whether the case of PAIS in question was AR‐related, this testing was not performed due to financial constraints. As a result, the diagnosis in this case is not conclusive and was made based on clinical, biochemical, karyotype, and radiological tests, treatment response, and family history. Another issue with the data for these patients is that androgen levels in prepubertal children are difficult to interpret due to the high variability in age and puberty status. Therefore, the endocrine results should be analyzed in connection with the phenotype and not solely used to make a diagnosis.

In conclusion, this case underscores the rare occurrence of PAIS in three members of a family, highlighting its complexity, resistance to exogenous androgen therapy, and the importance of multidisciplinary care. It also sheds light on the importance of early clinical diagnosis and genetic counseling to help plan reproductive and fertility‐related decisions.

## Author Contributions

**Tayyeb Ali:** validation, visualization, writing – original draft. **Muhammad Hassaan Javaid:** formal analysis, investigation, methodology, project administration. **Sibgha Fawad Memon:** conceptualization, data curation, formal analysis. **Zauha Fawad Memon:** software, supervision, validation. **Munazza Iqbal:** software, supervision, validation, writing – review and editing. **Sadia Afridi:** supervision, validation. **Abdul Rahim Soroush:** supervision, validation, visualization. **Adnan Iqbal Khan:** data curation, investigation, resources. **Naseer Ahmad:** resources, software. **Nayab Ayub Afridi:** visualization, writing – original draft. **Zuhaib Ali:** methodology, project administration.

## Funding

The authors have nothing to report.

## Disclosure

Written informed consent from all the patients' guardians was obtained for publication according to journal guidelines.

## Ethics Statement

Ethical Approval is not required for case series at our institution.

## Conflicts of Interest

The authors declare no conflicts of interest.

## Data Availability

The clinical data supporting the findings of this case series are available from the corresponding author upon reasonable request.
